# Osmolyte-Like Stabilizing Effects of Low GdnHCl Concentrations on d-Glucose/d-Galactose-Binding Protein

**DOI:** 10.3390/ijms18092008

**Published:** 2017-09-19

**Authors:** Alexander V. Fonin, Alexandra D. Golikova, Irina A. Zvereva, Sabato D’Auria, Maria Staiano, Vladimir N. Uversky, Irina M. Kuznetsova, Konstantin K. Turoverov

**Affiliations:** 1 Institute of Cytology of the Russian Academy of Sciences, Laboratory of Structural Dynamics, Stability and Folding of Proteins, Tikhoretsky av. 4, 194064 St. Petersburg, Russia; alexfonin@incras.ru (A.V.F.); imk@incras.ru (I.M.K.); 2 Saint Petersburg State University, Universitetskaya nab. 7/9, 199034 St. Petersburg, Russia; a.d.golikova@spbu.ru (A.D.G.); irina.zvereva@spbu.ru (I.A.Z.); 3 CNR, Institute of Food Science, via Roma 64, 83100 Avellino, Italy; sabato.dauria@cnr.it (S.D.); m.staiano@cnr.it (M.S.); 4 Department of Molecular Medicine and Byrd Alzheimer’s Research Institute, Morsani College of Medicine, University of South Florida, 12901 Bruce B. Downs Blvd., Tampa, FL 33612, USA; 5 Department of Biophysics, Peter the Great St. Petersburg Polytechnic University, Polytechnicheskaya av. 29, 195251 St. Petersburg, Russia

**Keywords:** d-glucose/d-galactose-binding protein, guanidine hydrochloride, osmolyte-like stabilizing effect, fluorescent label BADAN, protein conformers, protein function, conformational ensemble

## Abstract

The ability of d-glucose/d-galactose-binding protein (GGBP) to reversibly interact with its ligands, glucose and galactose, makes this protein an attractive candidate for sensing elements of glucose biosensors. This potential is largely responsible for attracting researchers to study the conformational properties of this protein. Previously, we showed that an increase in the fluorescence intensity of the fluorescent dye 6-bromoacetyl-2-dimetylaminonaphtalene (BADAN) is linked to the holo-form of the GGBP/H152C mutant in solutions containing sub-denaturing concentrations of guanidine hydrochloride (GdnHCl). It was hypothesized that low GdnHCl concentrations might lead to compaction of the protein, thereby facilitating ligand binding. In this work, we utilize BADAN fluorescence spectroscopy, intrinsic protein UV fluorescence spectroscopy, and isothermal titration calorimetry (ITC) to show that the sub-denaturing GdnHCl concentrations possess osmolyte-like stabilizing effects on the structural dynamics, conformational stability, and functional activity of GGBP/H152C and the wild type of this protein (wtGGBP). Our data are consistent with the model where low GdnHCl concentrations promote a shift in the dynamic distribution of the protein molecules toward a conformational ensemble enriched in molecules with a tighter structure and a more closed conformation. This promotes the increase in the configurational complementarity between the protein and glucose molecules that leads to the increase in glucose affinity in both GGBP/H152C and wtGGBP.

## 1. Introduction

Interactions of proteins with low-molecular weight ligands are crucial for the functioning of living systems. The most important biological processes, such as intracellular signaling, protein folding, catalysis, and the regulation of gene activities are controlled by such interactions. The binding of ligands to proteins can be accompanied by significant changes in protein conformation, and induced fit and conformational selection (population shift) models are often used for describing ligand–protein interaction. The induced fit model was proposed by Koshland [1] and is based on the assumption that ligand binding to protein induces conformational changes in a protein molecule. The conformational selection model suggests the existence in apo-form of an ensemble of protein conformers with different ligand binding affinities [2,3,4]. These conformers have comparable free energies [5], and ligands predominantly interact with a conformer, whose active center is the most complementary to the structure of the ligand [6]. This ensures that such an interaction is the most advantageous thermodynamically. According to this model, ligand binding to protein does not induce structural changes of the protein molecule, but causes the redistribution of the ensemble of protein conformers in solution. This model is some kind of ‘resuscitation’ of the classical ‘lock-and-key’ model proposed by Fischer [7] in 1894. In several studies, the interaction of some proteins with their ligands was described by a mixed mechanism, including elements of both conformational selection and induced fit models [8,9,10].

Periplasmic ligand-binding proteins (PBPs) are particularly interesting for studying functionally important structural changes in a protein molecule, since the conformations of the apo- and holo-forms of these proteins are typically rather different [11,12]. Proteins of this class are intermediary receptors of ABC transport systems and participate in the active transport of small soluble molecules (such as metal ions, amino acids, sugars, vitamins, and peptides) through cell membranes by hydrolysis of ATP [13]. PBPs consist of two domains connected by a hinge region, with an active center located within the cleft between the two domains [11]. The domains of these proteins can be considered as separate structural units with different mobilities that significantly changes on complex formation with ligands [14,15], and which have different relative locations in the apo- and holo-forms (open and closed conformers) [16]. For describing complex formation of PBPs with their ligands, both conformational selection and induced fit models are used. For example, the conformational selection mechanism was suggested for d-glucose/d-galactose-binding protein (GGBP) [17,18,19,20], ferric-binding protein (FBP) [21], and choline/acetylcholine substrate binding protein (ChoX) [22]. The induced fit mechanism was proposed for the description of the molecular mechanisms of action of ribose-binding protein (RBP) [18], glutamine-binding protein (GlnBP) [23], and maltose-binding protein (MBP) [24]. Furthermore, elements of both models were used in the description of the complex formation by l-lysine/l-arginine/l-ornithine-binding protein (LAO-BP) and MBP [25,26].

**Figure 1:**
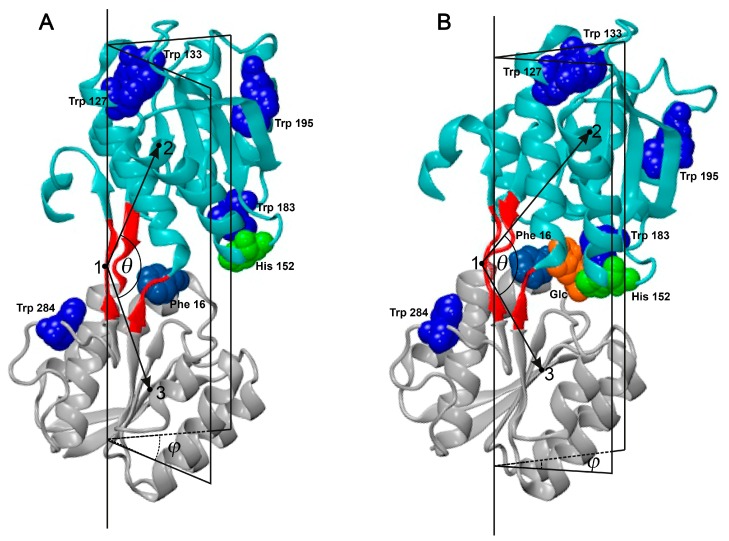
The 3D structure of d-glucose/d-galactose-binding protein (GGBP). Cartoon represents the 3D structure of GGBP constructed on the basis of X-ray data (PDB IDs: 2FW0 and 2FVY) [28]. N- and C-terminal domains are given in cyan and gray, protein hinge region is given in red. Tryptophan residues, Phe16, His152 (6-bromoacetyl-2-dimetylaminonaphtalene (BADAN) linked to position 152 when His is changed to Cys) and glucose are shown in space-filling diagram in blue, light blue, green, and orange, respectively. *θ* is an angle between the vectors directed from the mass center of the hinge region (point 1) to the mass centers of the C- and N-terminal domains (points 2 and 3). *φ* is a dihedral angle between the planes formed by strait line passing through two endpoints of the hinge region and the mass centers of the C- and N-terminal domains (points 2 and 3). Panel (**A**) represents structure of the apo-form of GGBP, whereas Panel (**B**) shows structure of the holo-form of this protein.

Similar to many other PBPs, GGBP consists of N- and C-terminal Rossmann fold domains linked by a hinge region [27] (Figure 1).

The GGBP active center is located in the cleft between these domains and is formed by residues Trp183 and Ala16, which, when stacked with sugar molecules, make the most dominant contribution to the interaction of this protein with a ligand [29,30].

GGBP interaction with its ligands (glucose and galactose) is characterized by very high affinities [18,31,32,33,34]. Hydrogen bonds between protein polar amino acid residues and sugar molecule significantly contribute to the fixation of the ligand within the GGBP active center [28,29]. Recently, the noticeable effect of water molecules on the specificity of glucose/galactose binding to GGBP was also shown [35].

Initially, it was assumed that GGBP molecules could only exist in two structural forms: open (without ligand or apo-form) and closed (with bounded ligand, or holo-form) [28,29,36]. Protein structures in open and closed forms differ by mutual arrangement of N- and C-terminal domains (Figure 1) and are described by the hinge angle (*θ*, the angle between the lines joining the mass centers of domains and the mass center of a hinge region) and the twist angles (*φ*, the dihedral angle formed by the hinge region of the protein and the half-planes passing through the mass centers of domains) [28,37]. Based on the results of fluorescent analysis, Messina and Talaga [38] suggested that in the absence of a ligand, a semi-closed, but not open, form of GGBP is dominant in the solution. In 2012, NMR-based analysis revealed that a solution with a GGBP structure is characterized by extensive segmental mobility, representing an ensemble of conformers, where the admissible values of angles *θ* and *φ* for GGBP apo- and holo-forms were largely overlapped [18]. Furthermore, it was pointed out that the highly flexible apo-form of this protein is characterized by an intermediate conformation that is structurally more related to the ligand-bound conformations, but transiently visits more open conformations, thereby enabling the selection of the most favorable conformation upon carbohydrate binding [18]. These data confirmed the early proposed model where the interaction of GGBP with its ligand can be described within the frames of the conformational selection mechanism [17,19,20]. However, in 2016, based on the paramagnetic NMR and molecular dynamic data, Unione et al. suggested a new model of the GGBP-glucose interaction, which combined elements of conformational selection and induced fit mechanisms [39]. According to this model, in the absence of the ligand, GGBP exists both in open and semi-closed conformations with an exchange rate of around 25 ns (conformational selection mechanism) [39]. Glucose injection promotes a shift in the dynamic conformational equilibrium from the open ligand-free form toward semi-closed conformations, which interacts with the glucose-forming closed conformation (induced fit mechanism) [39]. In addition to the significant fundamental value, these studies have indisputable practical value, since GGBP is considered as a promising candidate for sensing the element of glucose biosensors [40,41,42,43,44,45,46,47,48,49,50,51,52,53,54,55,56,57,58,59,60,61,62].

Previously, we found that the sub-denaturing concentrations of a widely used chemical denaturant, guanidine hydrochloride (GdnHCl), induced an increase in the fluorescence intensity of the fluorescent dye 6-bromoacetyl-2-dimetylaminonaphtalene (BADAN) linked to the glucose-bound form of the GGBP/H152C (which is a mutant protein form of GGBP with incorporated cysteine residue for covalent linking to BADAN) [63]. We explained this effect by an increase in the rigidity of the dye surround caused by the GGBP/H152C structure compaction [63] due to the so-called “stabilizing effect of low GdnHCl concentrations” [64,65,66,67,68,69,70,71,72,73,74]. However, we recorded no increase in the fluorescence intensity of BADAN linked to the GGBP/H152C apo-form [63]. The aim of the current work is to clarify whether the low GdnHCl concentrations can affect the apo-form of GGBP, and to examine whether the GdnHCl action on the GGBP structure can be regarded as a concentration-dependent effect of a sort of osmolyte that stabilizes proteins at low concentrations but destabilizes the protein structure at high concentrations [75].

## 2. Results and Discussion

GdnHCl is considered as one of the strongest denaturants, and in high concentrations can unfold (almost) any protein. Therefore, it is commonly used in physiochemical studies of protein folding. However, at low concentrations it has the quite opposite effect on protein structure, as was pointed out in several studies [64,65,66,67,68,69,70,71,72,73,74].

**Figure 2:**
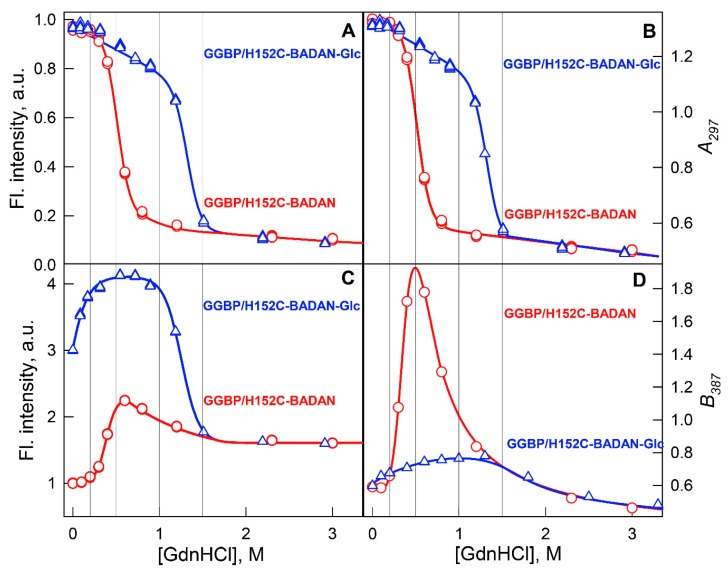
GdnHCl-induced conformational changes of GGBP/H152C–BADAN. Structural changes of protein apo-form (red curves and circles) and holo-form (blue curves and triangles) recorded by intrinsic UV fluorescence (Panels **A**,**B**) and by BADAN fluorescence (Panels **C**,**D**) are represented. The dependences of total intrinsic fluorescence intensity and total BADAN fluorescence intensity on the guanidine hydrochloride (GdnHCl) concentrations are represented on panels (**A**,**C**). Intrinsic fluorescence was excited at 297 nm, and BADAN fluorescence was excited at 387 nm. Shifts in the intrinsic and BADAN fluorescence spectrum position induced by GdnHCl are presented by parameters *A* and *B* (Panels **B**,**D**). Here *A* is a parameter for the intrinsic fluorescence spectrum position (*A* = *I*_320_/*I*_365_, where *I*_320_ and *I*_365_ are fluorescence intensities recorded at 320 and 365 nm, *λ*_ex_ = 297 nm), and *B* is a parameter for the BADAN fluorescence spectrum position (*B* = *I*_498_/*I*_587_, *I*_498_ and *I*_587_ are fluorescence intensities of BADAN recorded at 498 and 587 nm, *λ*_ex_ = 387 nm).

In this work, we analyzed the effect of GdnHCl on the wtGGBP and its mutant form GGBP/H152C. Earlier, we have shown that although four of five tryptophan residues (Trp127, Trp133, Trp183, and Trp195) are located in the C-terminal domain of GGBP, and although only one tryptophan (Trp284) is positioned within the N-terminal domain, the overall structural changes induced in this protein by GdnHCl can be recorded by its intrinsic fluorescence [76,77] (Figure 2A). At the same time, although one of the tryptophan residues (Trp183) is located within the glucose-binding center and is directly involved in sugar binding (Figure 1), glucose binding has little effect on GGBP intrinsic fluorescence [78]. Interestingly, interaction with glucose has a stronger effect on the local environments of other Trps than on the Trp183 environment [78]. However, the glucose binding can be easily assessed by changes in the fluorescence of the fluorescent probe BADAN covalently linked at position 152 of the GGBP/H152C mutant [47]. It turned out that the fluorescence of BADAN bound to this site is not only sensitive to the glucose binding, but also varies in a complex manner under the influence of GdnHCl (Figure 2C,D). Here, the fluorescence intensity of BADAN linked to the holo-form of GGBP/H152C first significantly increases in the range of 0.0–0.2 M GdnHCl, then remains unchanged in the range of 0.2–1.0 M GdnHCl, and finally dramatically decreases in the range of 1.0–1.5 M GdnHCl in parallel with the protein unfolding. In the case of apo-form, both the BADAN fluorescence intensity and the intensity of intrinsic fluorescence do not change at low GdnHCl concentrations (in the range of 0.0–0.2 M). In the range of GdnHCl concentrations where, according to intrinsic fluorescence, the protein unfolds (0.2–1.0 M), BADAN fluorescence intensity first increases, then decreases, and reaches the levels of BADAN fluorescence in the unfolded protein.

In the GdnHCl concentration range from 0.0 to 1.0 M, the position of the BADAN fluorescence spectrum, especially in the case of apo-form, changes significantly. In a solution of low GdnHCl concentrations (0.0–0.2 M), neither the position of the intrinsic fluorescence spectrum of GGBP nor the position of the spectrum of the BADAN changes. At the GdnHCl concentrations (0.2–1.0 M) where, according to intrinsic fluorescence, proteins unfold (red shift of fluorescence spectrum position), the red shift of the BADAN fluorescence spectrum is preceded by its significant blue shift. Therefore, the dependence of the BADAN fluorescence spectrum position on the GdnHCl concentration (Figure 2D, red curve) passes through the maximum for BADAN linked to the apo-form. However, there are no significant changes in the fluorescence spectrum position of the BADAN linked to the protein in the holo-form (Figure 2D, blue curve).

**Figure 3:**
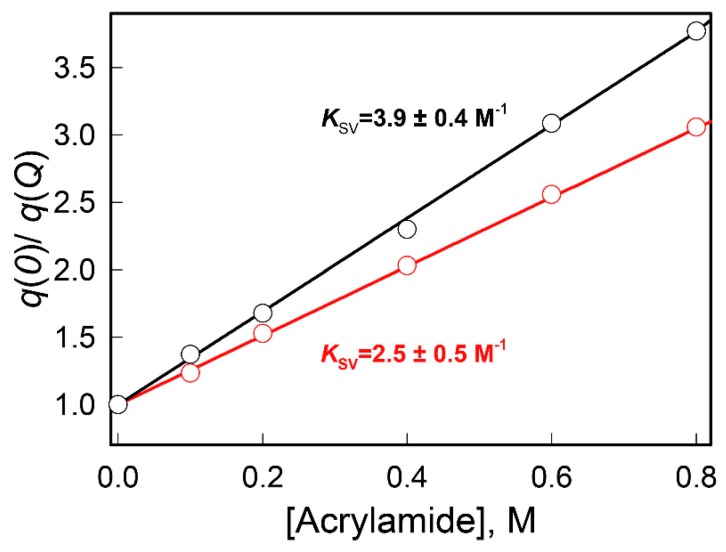
Acrylamide-induced quenching of intrinsic fluorescence of GGBP/H152C. Modified according Equation (12), Stern-Volmer dependences of the GGBP/H152C fluorescence intensity on acrylamide concentration in the solutions without denaturant (black curve) and in the presence of 0.1 M GdnHCl (red curve). The excitation wavelength was 297 nm.

These data suggest that low GdnHCl concentrations (0.0–0.2 M) cause compaction of the protein, which is manifested by an increase in the fluorescence intensity of BADAN linked to the holo-form. However, the intensity of BADAN fluorescence linked to the apo-form does not change much at similar conditions, since, in this case, the mobility of BADAN remains mostly unchanged. A significant increase in the compaction of GGBP/H152C induced by low GdnHCl concentration (0.1 M) was further demonstrated by the acrylamide-induced quenching of protein intrinsic fluorescence (Figure 3).

Noticeable changes in the fluorescence characteristics of BADAN linked to the GGBP/H152C apo-form are observed at higher GdnHCl concentrations (0.2–0.5 M). An increase in the fluorescence intensity, and a blue shift in the fluorescence spectrum position, would seem to reflect an increase in the rigidity of the BADAN microenvironment under these conditions. However, an increase in the amplitude of the high-frequency oscillations of the BADAN linked to the GGBP/H152C (Table 1) does not support this hypothesis.

Apparently, the recorded changes in the fluorescence characteristics of BADAN linked to GGBP/H152C in the presence of these concentrations of GdnHCl are due to the significant changes in the solvation of the dye [79]. The absence of noticeable changes in the intensity and the position of the BADAN fluorescence spectrum linked to the holo-form in this range of the GdnHCl concentrations can be explained by the fact that, in this case, BADAN is already in a sufficiently rigid environment and is largely protected from the solvent.

**Figure 4:**
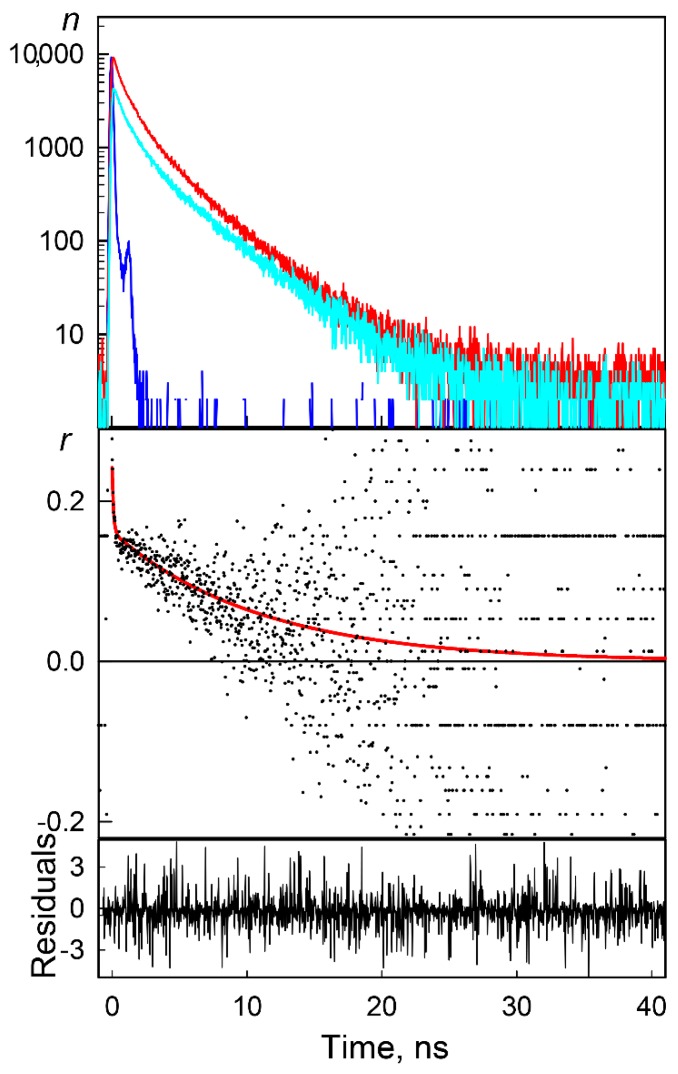
Time-resolved fluorescence anisotropy of BADAN linked to GGBP/H152C. The top panel represents the experimental decay curves of parallel (red curve) and perpendicular (cyan curve) components of the BADAN fluorescence; the instrument response function (blue curve) in semi-logarithmic scale. The middle panel represents time-resolved anisotropy decay calculated according to Equation (3) (dots) and the best fit (red curve); weighted residuals are shown at the bottom panel.

The increase in the rigidity of the microenvironment of BADAN linked to the holo-form was confirmed by time domain experiments on fluorescence anisotropy. We recorded the fluorescence decay curves of BADAN linked to the apo- and holo-form of the protein in planes parallel and perpendicular to the plane of the exciting light. As an example, Figure 4 represents the measured decay curves measured for the parallel and perpendicular to the excitation light components of the BADAN fluorescence linked to the protein apo-form in the absence of a denaturant, as well as the time course of the calculated fluorescence anisotropy. It is obvious that the time dependence of fluorescence anisotropy can be described by a bi-exponential decay, which may indicate the presence of both fast and slow mobility of BADAN. The kinetic dependences of the BADAN fluorescence anisotropy were measured for the apo-and holo-form GGBP/H152C in the absence and presence of GdnHCl at concentrations of 0.1 and 0.5 M (Table 1).

Anisotropy decay curves were approximated by bi-exponential functions. This allows the separation of the ‘slow’ and ‘fast’ BADAN motions and the calculation of the mean amplitude of dye high frequency mobility, *θ*. It was shown that the intramolecular mobility of BADAN is restricted by the complex formation of GGBP/H152C with glucose. Sufficient restriction of the dye internal motion also was observed in solutions with GdnHCl sub-denaturing concentrations for glucose-bound GGBP/H152C (Table 1). The most noticeable effect was found for the GGBP/H152C holo-form in the solution of 0.5 M GdnHCl (Table 1). These data supported our hypothesis that the increase in the fluorescence intensity of BADAN linked to the GGBP/H152C holo-form in solutions with sub-denaturing GdnHCl concentrations is caused by the increase in the rigidity of the BADAN local environment.

**Figure 5:**
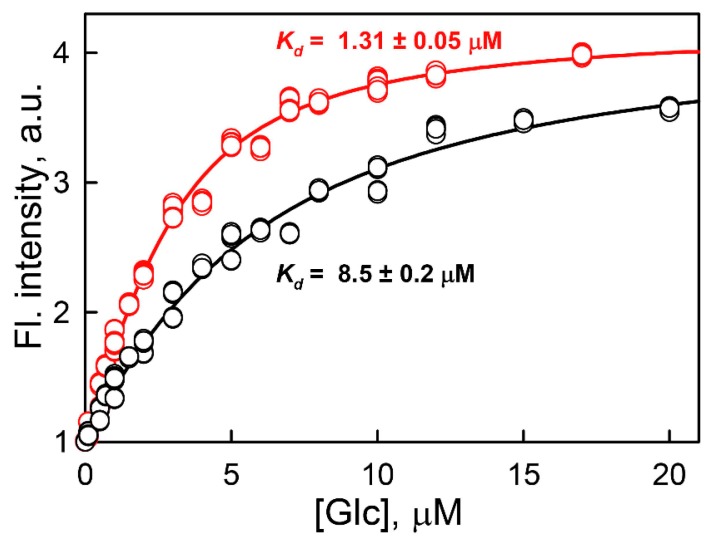
Dependence of the BADAN fluorescence intensity linked to GGBP/H152C on glucose concentrations [Glc] in solution without denaturants (black curve) and in the presence of 0.1 M GdnHCl (red curve). The excitation and emission wavelengths were 387 and 545 nm, respectively.

It is known that protecting osmolytes contribute not only to the formation of a rigid globular structure but also to the functional activity of the protein [80,81,82,83,84,85,86,87]. Therefore, we examined the affinity of glucose to GGBP/H152C in solutions with sub-denaturing GdnHCl concentrations. It was found that, in the presence of 0.1 M GdnHCl, the dissociation constant was significantly lower (*K_d_* = 1.31 ± 0.05 μM) than that measured in the absence of denaturant (*K_d_* = 8.5 ± 0.3 μM) (Figure 5). This proves the idea that in solutions with sub-denaturing GdnHCl concentrations the affinity of glucose to GGBP/H152C increases, due to the shift of the dynamic distribution of the GGBP/H152C molecules in apo-form to molecules with more closed conformation and tighter structure.

The greater affinity of GGBP to glucose in the presence of low GdnHCl concentrations was also manifested by an increase in the binding rate of glucose to the protein in the presence of 0.1 M GdnHCl. It is known that, at high concentrations of one of the reactants, the reaction rate is predominately determined by the content of the substance with a low concentration (pseudo-first order conditions) [88,89,90]. Therefore, it can be expected that in solutions of high glucose concentration, the rate of the GGBP/H152C-glucose complex formation will be determined by the content of molecules with the semi-closed conformations in a protein ensemble. The kinetics of the glucose binding to GGBP/H152C at the ligand excess conditions were studied by the stopped-flow technique in solutions without denaturant and in the presence of sub-denaturing GdnHCl concentrations.

**Figure 6:**
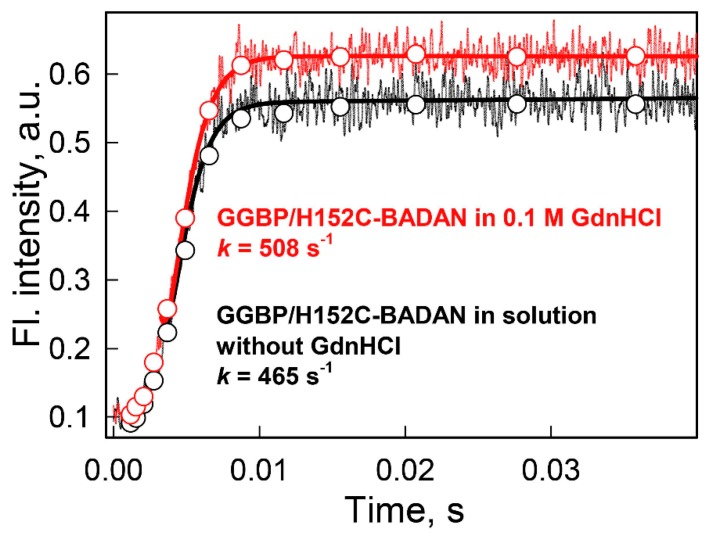
The kinetics of glucose binding to GGBP/H152C-BADAN in solutions without denaturants (black curve) and in the presence of 0.1 MGdnHCl (red curve). The solid lines represent best fit of data by exponential model. The circles represent the data array after logarithmic transformation of experimental data. The excitation wavelength was 387 nm. Glucose concentration was 0.5 mM.

It is expected that the decay curves should contain at least two rate constants characterizing the interconversion between the protein conformers in the absence of ligand and reflecting the glucose binding to GGBP/H152C [88]. However, the exchange rate between different conformations of GGBP apo-form is ~25 ns [39], which is substantially shorter than the dead-time of the stopped-flow instrument. Therefore, in practice, the recorded curves are approximated by monoexponential decay. This analysis revealed that the formation of the GGBP/H152C-glucose complex occurred somewhat faster in solutions with the sub-denaturing GdnHCl concentrations (*k* = 508 s^−1^ in 0.1 M GdnHCl) in comparison with the complex formation in the absence of denaturants (*k* = 465 s^−1^) (Figure 6). These data also confirmed the shift of the conformational ensemble of the GGBP/H152C apo-form to molecules with more closed conformations and tighter structure induced by the GdnHCl addition at the sub-denaturing concentrations.

**Figure 7:**
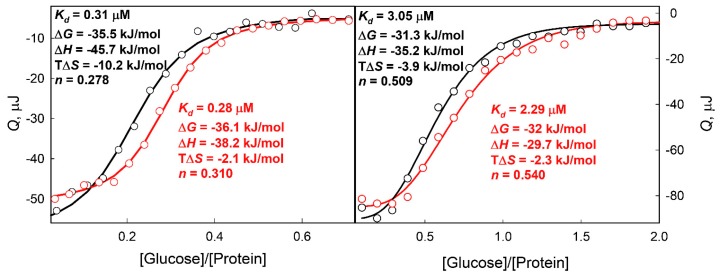
Isothermal calorimetric titration (ITC) analysis of the wtGGBP and GGBP/H152C interaction with glucose. ITC profiles for wtGGBP (left panel) and GGBP/H152C (right panel) in solution without denaturants (black circles) and in the presence of 0.1 M GdnHCl (red circles) are shown. The curves represent best fit of experimental data by the independent binding sites model. The glucose initial concentrations were 0.25 mM (for wtGGBP) and 0.7 mM (for GGBP/H152C). The initial protein concentrations were 0.035 mM.

To show that the increase in glucose affinity to GGBP/H152C-BADAN in solutions with low GdnHCl concentrations is not a result of the specific interaction of GdnHCl with BADAN or the influence of the His152Cys amino acid replacement, the effects of low GdnHCl concentrations on the affinity of glucose to the wtGGBP and GGBP/H152C were studied by label-free approaches. For this purpose, we used isothermal titration calorimetry (ITC) (Figure 7), since this approach allows for the determination of both the stoichiometric and thermodynamic binding parameters [91]. The results of the ITC analysis generally corresponded to the data generated by the fluorescent-based analysis of the GGBP/H152C-BADAN interaction with glucose in solutions with sub-denaturing GdnHCl concentrations. The highest affinity of glucose to the GGBP/H152C and wtGGBP was observed in solutions with the sub-denaturing GdnHCl concentrations (*K_d_* = 0.28 ± 0.06 μM for wtGGBP and *K_d_* = 2.29 ± 0.08 μM for GGBP/H152C in 0.1 M GdnHCl) (Figure 7). The enthalpy contribution to the Gibbs free energy change caused by the interaction of studied proteins with glucose was principal in all solutions tested. This indicates the significant contribution of the hydrogen bonds between protein and glucose to the protein-ligand complex formation, [91] which is in excellent agreement with the X-ray structural data showing that the glucose binding to the GGBP active center is stabilized by 13 hydrogen bonds [28,29]. The entropy of the considered systems of a protein interacting with glucose was changed less in the solutions with the sub-denaturing GdnHCl concentrations than in the solutions without denaturants. Therefore, the macrostate of the considered system (population of all protein molecules) in the sub-denaturing GdnHCl concentrations is realized by a smaller number of microstates (GGBP conformations) than in other studied solutions.

In other words, the distribution of the protein conformers of GGBP and GGBP/H152C interacting with glucose is changed less in solutions with the sub-denaturing GdnHCl concentrations than in other tested solutions. The stoichiometry of glucose binding to the wtGGBP and GGBP/H152C is significantly less than unity, confirming the participation of proteins with only semi-closed conformation in sugar binding [92]. According to Unione et al., about 30% of GGBP molecules have semi-closed conformation when this protein exists in an apo-form [39]. It should be noted that some difference in the parameters of glucose binding to GGBP/H152C obtained by BADAN fluorescence spectroscopy and ITC are probably due to the steric effect of the dye on the interaction of protein with glucose.

Taken together, our findings conclude that GdnHCl in sub-denaturing concentrations promotes the narrowing of the dynamic distribution of the molecules of the wtGGBP and GGBP/H152C in the absence of glucose, shifting it to the molecules with a more closed conformation and a tighter structure. This induces an increase in glucose affinity to a protein, as the structure of the protein active center in a semi-closed conformation better complements the glucose structure than the structure of the protein active center in an open conformation.

In 1996, Butler and Falke used disulfide trapping to show significant inhibition of the large amplitude motions of GGBP, including the interdomain motions in solutions of some osmolytes [93]. It is known that the amplitude of the slow, large-scale mobility of proteins depends significantly on backbone solvation [94]. The stabilizing/destabilizing effects of osmolytes on proteins is also predominantly determined by protein solvation [95,96]. Considering GdnHCl as an osmolyte-like concentration-dependent agent, it can be assumed that low GdnHCl concentrations induce significant changes in the GGBP solvation. By analogy, with the effect of osmolytes on GGBP structural dynamics [93], it can be also hypothesized that the protein large amplitude motions, including the interdomain dynamics, are restricted in the solutions with low GdnHCl concentrations. Unione et al. showed a high conformational mobility of GGBP in apo-form and a significant restriction of the interdomain dynamics associated with the protein complex formation with glucose [39]. Therefore, it is likely that the restriction of the GGBP large amplitude motions induced by low GdnHCl concentrations could induce a shift from the more open ligand-free forms to a more closed conformation, and thereby cause an increase in the glucose affinity to GGBP.

The results obtained in this study have not only fundamental but also practical significance. As was already mentioned, PBPs are actively used in the development of the sensing elements of biosensor systems for the detection of various analytes [13,40,41,42,43,44,45,46,47,48,49,50,51,52,53,54,55,56,57,58,59,60,61,62,97]. In order for the biosensor system to continuously respond to changes in the concentration of the testing substance in an analyzed medium, the dissociation constant of a sensing element-analyte complex should correspond to the middle of the tested range of the analyte concentrations. In practice, the analyte concentration ranges could significantly differ (sometimes by orders of magnitude) from the values of the dissociation constant of the sensing element-analyte complex. The most popular method for solving this problem is the creation of protein mutant forms (sensing elements) possessing a different affinity to the ligand. Amino acid residues in a protein active center are usually chosen as mutagenesis targets. In the vast majority of cases, the resulting mutant forms of ligand-binding proteins have poorer affinity to the ligand in comparison with wild type proteins. This may impair the selectivity of analyte-protein binding and induce other problems [98]. However, the development of the proteinaceous sensing elements of the biosensor systems based on the ligand-binding proteins often requires the generation of PBP mutant forms with an increased/decreased affinity to the ligand in comparison with the corresponding wild type proteins. To achieve this, the creation of the PBP mutant forms with amino acid replacements in the hinge region was proposed, which are involved in the allosteric regulation of the mutual arrangement of the protein domains [97,99,100,101].

In our opinion, the wider use of PBPs in the development of sensing elements of the biosensor systems and in the drug design requires special means to control the conformation of ligand-binding proteins without disrupting the structures of their active centers. This can be achieved based not only on the currently utilized genetic engineering approaches, but also on a better understanding of how to control protein interactivity through changes in solvent properties.

## 3. Materials and Methods

### 3.1. Materials

d-Glucose, guanidine hydrochloride, urea, tris(2-carboxyethylphosphine (TCEP), acrylamide (Sigma, St. Louis, MO, USA), and fluorescent label BADAN (AnaSpec, Fremont, CA, USA) were used without further purification. To determine the GdnHCl concentration, we relied on the measurement of the refraction coefficient using the Abbe refractometer (LOMO, St. Petersburg, Russia).

*E. coli* strain K-12 (*F^+^mgl503lacZlacY*^+^*recA1*) carrying an *mglB* gene deletion [59,102] and transformed with a pTz18u-*mglB* vector was primarily used for obtaining the wtGGBP. Upon induction with d-fructose [103], the expression efficiency of the GGBP protein was rather low: the recombinant protein yield in this system did not exceed 5–8 mg/L of culture. Therefore, to increase the expression levels, the nucleotide sequence of the *mglB* gene was optimized and the gene was recloned into a pET-11d plasmid with the T7 promoter (Stratagene, La Jolla, CA, USA) using the *Nco I-BamH I* and *Bgl II* restriction sites. Specific forward and reverse primers were used to insert new restriction sites and a polyhistidine tag at the C-terminal of the gene. Site-directed mutagenesis was performed with a Quik-Change mutagenesis kit (Stratagene, La Jolla, CA, USA) using primers encoded for the corresponding amino acid substitutions. Plasmids were isolated from bacterial cells using plasmid DNA isolation kits (Omnix, St. Petersburg, Russia). Primer purification was performed by either reverse-phase chromatography or electrophoresis in a polyacrylamide gel.

pET-11d plasmids encoding for the wtGGBP and GGBP/H152C mutant were used to transform *E. coli* BL21(DE3) cells. The expression of the proteins was then induced by adding 0.5 mM isopropyl-beta-d-1-thiogalactopyranoside (IPTG; Nacalai Tesque, Kyoto, Japan). Bacterial cells were cultured for 24 h at 37 °C. Recombinant proteins were purified using Ni^++^-agarose packed in His-GraviTrap columns (GE Healthcare, Chicago, IL, USA). Protein purification was controlled using denaturing SDS-electrophoresis in 15% polyacrylamide gel [104].

The labeling of GGBP/H152C with the fluorescent dye BADAN was performed as described by Khan [49] with a slight modification. To label proteins with BADAN, a 100-fold excess of TCEP was added in solution, and a 10-fold excess of dye was added to the obtained mixture. The resulting reaction mixture was incubated overnight at 4 °C. The unbound dye was removed by filtration along with an extensive dialysis against a sodium phosphate buffer.

The experiments were performed in protein solutions with concentration of 0.2 mg/mL. For the formation of the protein-ligand complex, 0.05 μM–20mM of d-glucose was added to the protein solution. All measurements were conducted in the sodium phosphate buffer, pH 7.4. All experiments were performed at 23 °C.

### 3.2. Methods

#### 3.2.1. Steady-State Fluorescence Spectroscopy

The fluorescence experiments were carried out using a Cary Eclipse (Agilent, Santa Clara, CA, USA) spectrofluorimeter. The measurements were made at 23 °C using 10 × 10 mm cells (Starna, Atascadero, CA, USA, USA). The fluorescence intensity of BADAN and tryptophan residues was corrected for the primary inner filter effect [105]:

(1) $$F_{0}\left(\lambda_{ex}\right)=F\left(\lambda_{ex}\right)/W$$

where *W* is factor which corrects measured total fluorescence intensity for the so-called primary inner filter effect, and *F*(*λ_ex_*) is a total fluorescence intensity.

Because the fluorescence measurements were performed using the Cary Eclipse spectrofluorimeter with horizontal slits, the value of correction factor *W* was calculated based on the following ratio:

(2) $$W=\frac{\left(1-10^{-A_{\Sigma }}\right)}{A_{\Sigma }}$$

where *A*_Σ_ is the total absorbance of exciting light in the solution. Absorption spectra were registered using a U-3900H (Hitachi, Tokyo, Japan) spectrophotometer. Earlier [105], it was shown that the value of the total fluorescence intensity corrected in such a manner is proportional to the product of the absorbance *A_FL_* to the quantum yield of fluorescence *q*, when one fluorescent substance is in present solution.

The excitation wavelength for the intrinsic protein fluorescence was 297 nm, whereas the emission wavelengths for detecting the intrinsic protein fluorescence ranged from 300 to 450 nm. The dye fluorescence was excited at 387 nm. The emission wavelength for the BADAN fluorescence was ranged from 400 to 650 nm.

Fluorescence anisotropy was determined as:

(3) $$r=\frac{\left(I_{V}^{V}-GI_{H}^{V}\right)}{\left(I_{V}^{V}+2GI_{H}^{V}\right)}$$

where $I_{V}^{V}$ and $I_{H}^{V}$ are vertical and horizontal components of fluorescence intensity excited by vertical polarized light, and $G={I_{V}^{V}/I}_{H}^{H}$ is the coefficient that determines the different sensitivity of the registering system for the vertical and horizontal components of the fluorescence light.

The fluorescence intensity of the protein solution in the presence of ligand (assuming that there are two forms of a protein in solution) can be determined by the following equation:

(4) $$F(C_{0})=\alpha_{F}(C_{0})F_{F}+\alpha_{B}(C_{0})F_{B}$$

where *F_F_* and *F_B_* are the protein fluorescence intensity in free state and in a ligand-bound form, respectively, and *α_F_*(*C*_0_) and *α_B_*(*C*_0_) are the relative fractions of the corresponding protein states in the solution at a concentration of added ligand *C*_0_, *α_F_*(*C*_0_) + *α_B_*(*C*_0_) = 1. Thus, the fraction of bound protein is determined as:

(5) $$\alpha_{B}=\frac{F(C_{0})-F_{F}}{F_{B}-F_{F}}=\frac{C_{b}}{C_{p}}$$

where *C_p_* is the total protein concentration and *C_b_* is the concentration of protein bound to ligand. The dissociation constant, *K_d_*, can be expressed as follows:

(6) $$K_{d}=\frac{\left[receptor\right]\cdot \left[ligand\right]}{\left[complex\right]}=\frac{(C_{p}-C_{b})\cdot C_{f}}{C_{b}}$$

where *C_f_* is the concentration of the free ligand, which can be calculated from the equation:

(7) $$C_{b}=C_{0}-C_{f}$$

Eliminating *C_f_* from Equation (6), we can obtain the following expression for *C_b_*:

(8) $$C_{b}=\frac{(K_{d}+C_{p}+C_{0})-\sqrt{{\left(K_{d}+C_{p}+C_{0}\right)}^{2}-4C_{p}\cdot C_{0}}}{2}$$

Combining Equations (5) and (8), the equation for the definition of *K_d_* can be derived using the difference in fluorescence intensity of a protein in the presence or absence of glucose:

(9) $$F(C_{0})=F_{F}+(F_{B}-F_{F})\cdot \frac{\left(K_{d}+C_{p}+C_{0}\right)-\sqrt{{\left(K_{d}+C_{p}+C_{0}\right)}^{2}-4C_{p}\cdot C_{0}}}{2C_{p}}$$

The approximation of experimental data was performed via the nonlinear regression method using the Sigma Plot program.

#### 3.2.2. Time-Resolved Fluorescence Anisotropy Spectroscopy

Time-resolved fluorescence measurements were carried out by a time-correlated single-photon counting approach using the Fluotime 300 (Pico Quant, Berlin, Germany) spectrometer with the Laser Diode Head LDH-375 (*λ*_ex_ = 375 nm).

The fluorescence anisotropy decay was obtained using Equation (3) and recording $I_{V}^{V}(t)$ and $I_{H}^{V}(t)$ at magic angle conditions. The *G* coefficient for Fluotime 300 was calculated for every measurement and was approximately equal to 1. The anisotropy decays were fit using the two component approximation:

(10) $$r(t)=\sum_{i}^{2}r_{i}\cdot e^{-t/\varphi_{i}}$$

where *r_i_* is the anisotropy of *i*th component, and *φ_i_* is the rotation correlation time of the *i*th component. According to this approximation, a decrease in the fluorescence anisotropy of BADAN linked to GGBP/H152C represents a sum of “slow” and “fast” motions of this dye [106]. The “slow” motion corresponds to the rotation of a protein as a whole with the rotation relaxation time *τ_slow_* = 3 *ϕ_slow_*, whereas the “fast” motion corresponds to the rotation of a dye with the rotation relaxation time *τ_fast_* = 3 *ϕ_fast_*. The anisotropy profiles allow the calculation of the mean amplitude of the dye motions, *θ*, within the free oscillation model of a fluorophore in a discrete potential hole with a width of *θ* as follows [107,108]:

(11) $$\frac{r_{fast}}{r_{0}}=1-\frac{\cos^{2}\theta {\left(1+\cos\theta \right)}^{2}}{4}$$

where $r_{0}=r\left(t=0\right)=r_{fast}+r_{slow}$.

The convolution of the model exponential function with the instrument response function (IRF) was compared to the experimental data until a satisfactory fit was obtained. The IRF was measured using the cross correlation of the excitation and fundamental gate pulse. The FluoFit software (Pico Quant, Berlin, Germany, version 4.6.6) was used for the analysis of the decay curves.

#### 3.2.3. The Dynamic Quenching of the Intrinsic UV Fluorescence

The study of the dynamic quenching of the wild type GGBP intrinsic UV fluorescence by the low molecular quencher acrylamide in solution without denaturants and in 0.1 M GdnHCl was carried out for evaluating the accessibility of the protein tryptophan residues to solvent in these conditions. The protein intrinsic fluorescence was excited at 297 nm and the total intrinsic UV fluorescence (from 300 to 450 nm) was registered. It is worth mentioning that the acrylamide absorbs at the wavelength of excitation 297 nm. Therefore, the recorded fluorescence intensity should be amended, relating to changes in *W* with the increase of quencher concentration.

The quenching constants were obtained using the Stern-Volmer equation written for total fluorescence intensity:

(12) $$\frac{F(0)}{F(Q)}=\frac{W(0)}{W(Q)}\left(1+K_{SV}Q\right)$$

where *F*(0) is the total fluorescence in the absence of quencher, *F*(*Q*) is the total fluorescence in the presence of quencher at a concentration *Q*, *W*(0) is the correction factor *W* in the absence of quencher, *W*(*Q*) is the correction factor *W* in the presence of quencher, *K_SV_* is Stern-Volmer constant, i.e., the rate constant of fluorescence quenching due to collisions of quencher molecules with fluorophore molecules in the excited state. Here it is accepted that *A_FL_*(0) = *A_FL_*(*Q*).

#### 3.2.4. Stopped-Flow Fluorescence Kinetic Experiments

The kinetics of glucose binding to GGBP/H152C-BADAN in a timeframe from 0 to 0.04 s was measured using the MOS450spectrometerequipped with the stopped-flow mixing module SFM-4 (Bio-Logic, Seyssinet-Pariset, France). The excitation wavelength was 387 nm, the dye fluorescence signal was filtered by cut-off filter at 405 nm. The PMT voltage was 900 V. In all experiments, an FC-15 cuvette with a path length of 1.5 mm was used; the total flow rate was 14 mL/s, and the dead time of apparatus was 3 ms. The final concentrations after mixing were 0.6 μM for GGBP/H152C-BADAN and 0.5 mM for glucose in a corresponding buffer (sodium phosphate buffer or 0.1 M GdnHCl in sodium phosphate buffer). All the kinetic experiments were repeated 30–40 times to decrease the ratio of noise to useful signal. The temperature of the syringes, mixers, flow cell, and the connecting lines was maintained constant at 23 °C with a circulating bath. The analysis of the kinetics data was carried out by Bio-Kine software (Bio-Logic) according to the conformational selection model. This model assumes that only the semi-closed apo-form of GGBP/H152C can interact with glucose:

(13) $$E\overset{k_{1}}{\underset{k_{2}}{⇄}}E*+L\overset{k_{3}}{\underset{k_{4}}{⇄}}EL$$

where *E* is the GGBP/H152C open apo-form, E* is the GGBP/H152C semi-closed apo-form, *L* is the glucose, and *EL* is the complex of GGBP/H152C with glucose. In this case, the observed kinetic curve is characterized by a bi-exponential function with constants [89]:

(14) $$k_{obs1,2}=\frac{k_{2}+k_{1}+k_{4}+k_{3}[L]\pm \sqrt{{\left(k_{4}+k_{3}[L]-k_{2}-k_{1}\right)}^{2}+4k_{2}k_{3}[L]}}{2}$$

Assuming that [E*] << [L], the relation (14) takes the following forms [89]:

(15) $$\begin{array}{l} k_{obs1}=k_{3}[L]\\ k_{obs2}=k_{1} \end{array}$$

#### 3.2.5. Isothermal Titration Calorimetry

The parameters of the binding of mutant GGBP/H152C and wtGGBP with glucose in buffer, GdnHCl, and urea solutions were determined using the isothermal titration calorimeter TA Nano ITC (TA Instruments, New Castle, DE, USA) at the Resource Center of the Thermogravimetric and Calorimetric Research (Research Park of St. Petersburg State University). Protein concentration was 35 μM in the final sample volume of 1 mL. The reference cell was filled with corresponding buffer (sodium phosphate buffer or 0.1 M GdnHCl in sodium phosphate buffer). Twenty injections (4.91 μL each) of 0.25 mM glucose for the wtGGBP and 0.7 mM glucose for GGBP/H152C in the corresponding buffer were carried out during every experiment. The interval between injections was 300 s for baseline stabilization. The stirring speed was constant and equal to 300 rpm. The results for the first injection were discarded due to the diffusion in the tip of the syringe during baseline stabilization. The titration of buffer by buffer was carried out for checking the thermostatic control of the cell and syringe. It was established that during such titration, heat effects larger than noise levels were not observed, confirming the temperature equality of titrant and analyte. The analysis of the ITC data was performed by NanoAnalyse software (TA Instruments) using an independent binding model based on the Wiseman isotherm [109,110,111]:

(16) $$Q=\frac{n{\left[M\right]}_{t}V_{0}\Delta H}{2}\left(1+\frac{{\left[X\right]}_{t}}{n{\left[M\right]}_{t}}+\frac{K_{d}}{n{\left[M\right]}_{t}}-\sqrt{{\left(1+\frac{{\left[X\right]}_{t}}{n{\left[M\right]}_{t}}+\frac{K_{d}}{n{\left[M\right]}_{t}}\right)}^{2}-\frac{4{\left[X\right]}_{t}}{n{\left[M\right]}_{t}}}\right)$$

where *Q* is the total heat content of solution, *V*_0_ is the volume of calorimeter cell, ∆*H* is the change of enthalpy of binding, *n* is the number of binding sites per macromolecule, *K_d_* is the dissociation constant, and [*X*]*_t_* and [*M*]*_t_* are the total concentration of ligand and macromolecule in calorimeter cell, which are equal to

(17) $$\begin{array}{l} {\left[X\right]}_{t}={\left[X\right]}_{0}\left(1-\left(1-\frac{v}{V_{0}}\right)\right)\\ {\left[M\right]}_{t}={\left[M\right]}_{0}\left(1-\left(1-\frac{v}{V_{0}}\right)\right) \end{array}$$

where [*X*]_0_ and [*M*]_0_ are the initial concentration of ligand and macromolecule, and *v* is the injection volume.

## 4. Conclusions

In this study, we utilized BADAN fluorescence spectroscopy, intrinsic UV fluorescence spectroscopy, and isothermal titration spectroscopy (ITC) to show that the sub-denaturing GdnHCl concentrations affect the dynamic conformational equilibrium of the GGBP molecules. Namely, at the sub-denaturing concentrations, GdnHCl promotes a shift in the dynamic distribution of protein molecules toward the conformational ensemble-containing molecules with a tighter structure and a more closed conformation. As glucose molecules have a higher affinity to more closed conformations of GGBP, sub-denaturing GdnHCl concentrations also caused the enhancement of the GGBP activity. Consequently, it can be concluded that low GdnHCl concentrations have an osmolyte-like stabilizing effect on GGBP. Since the further increase in the GdnHCl concentration leads to protein denaturation and unfolding, GdnHCl can be regarded (at least in relation to GGBP) as a concentration-dependent protecting osmolyte.

## Figures and Tables

**Table 1:** The time-resolved characteristics of anisotropy fluorescence of BADAN linked to the GGBP/H152C in solutions with the sub-denaturing GdnHCl concentrations *.

**GGBP/H152C-BADAN Apo-Form**								
**GdnHCl Concentration, M**	**<*t*>, ns**	***r_fast_***	***r_slow_***	***r*_0_**	*θ* ** Degree	***τ_fast_*, ns**	***τ_slow_*, ns**	***χ*^2^**
0.0	1.3	0.08	0.16	0.24	29	0.3	34	0.87
0.1	1.4	0.08	0.19	0.27	28	0.4	30	0.99
0.5	2.7	0.14	0.14	0.28	38	0.3	30	1.08
**GGBP/H152C-BADAN Holo-Form**								
**GdnHCl Concentration, M**	**<*t*>, ns**	***r_fast_***	***r_slow_***	***r*_0_**	*θ* **, Degree	***τ_fast_*, ns**	***τ_slow_*, ns**	***χ*^2^**
0.0	3.1	0.07	0.19	0.26	25	0.3	33	1.01
0.1	3.1	0.04	0.16	0.20	22	1.5	27	1.06
0.5	3.4	0.04	0.25	0.29	19	1.2	28	1.10

* The errors in calculated parameters of time-resolved anisotropy fluorescence lie within 5–10%. ** The mean amplitude of the dye motions, *θ*, was calculated according to the Equation (11).
